# Axon mimicking hydrophilic hollow polycaprolactone microfibres for diffusion magnetic resonance imaging

**DOI:** 10.1016/j.matdes.2017.10.047

**Published:** 2018-01-05

**Authors:** Feng-Lei Zhou, Zhanxiong Li, Julie E. Gough, Penny L. Hubbard Cristinacce, Geoff J.M. Parker

**Affiliations:** a Division of Informatics, Imaging and Data Sciences, The University of Manchester, Manchester M13 9PT, United Kingdom; b CRUK and EPSRC Cancer Imaging Centre in Cambridge and Manchester, UK; c The School of Materials, The University of Manchester, Manchester M13 9PL, United Kingdom; d College of Textile and Clothing Engineering, Soochow University, Suzhou 215000, PR China; e School of Psychological Sciences, The University of Manchester, Manchester M13 9PT, United Kingdom; f Bioxydyn Limited, Rutherford House, Manchester Science Park, Pencroft Way, Manchester M15 6SZ, United Kingdom

**Keywords:** Axon mimic, Polycaprolactone, Hydrophilic hollow microfibres, Co-electrospinning, Diffusion magnetic resonance imaging

## Abstract

Highly hydrophilic hollow polycaprolactone (PCL) microfibres were developed as building elements to create tissue-mimicking test objects (phantoms) for validation of diffusion magnetic resonance imaging (MRI). These microfibres were fabricated by the co-electrospinning of PCL-polysiloxane-based surfactant (PSi) mixture as shell and polyethylene oxide as core. The addition of PSi had a significant effect on the size of resultant electrospun fibres and the formation of hollow microfibres. The presence of PSi in both co-electrospun PCL microfibre surface and cross-section, revealed by X-ray energy dispersive spectroscopy (EDX), enabled water to wet these fibres completely (i.e., zero contact angle) and remained active for up to 12 months after immersing in water. PCL and PCL-PSi fibres with uniaxial orientation were constructed into water-filled phantoms. MR measurement revealed that water molecules diffuse anisotropically in the PCL-PSi phantom. Co-electrospun hollow PCL-PSi microfibres have desirable hydrophilic properties for the construction of a new generation of tissue-mimicking dMRI phantoms.

## Highlights

• An inclusion of polysiloxane-based surfactant (PSi) into PCL solution improved the hydrophilicity of electrospun PCL fibres.
• Electrospun PCL-PSi fibres remained hydrophilic over a time period of up to 12 months.
• EDX analysis confirmed Si distribution on fibre surface and cross-section, which is responsible for the wettability of PCL-PSi fibres.
• Hollow PCL-PSi microfibres were only produced via co-electrospinning of a miscible combination of shell and core solutions.
• Phantoms were constructed from hollow PCL and PCL-PSi fibres, and water was detected by dMRI only from the latter.

## Graphical abstract

**Figure:**
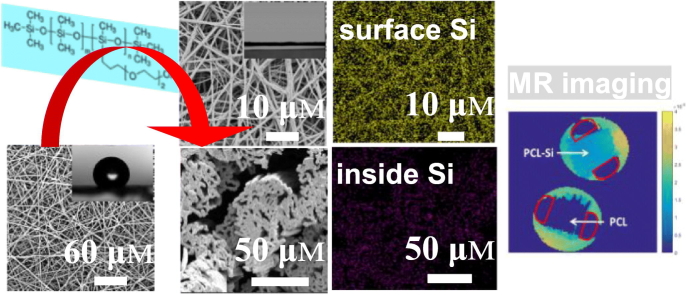

## 1 Introduction

The mobility of water molecules within tissue depends on the microstructure of the tissue. Brain white matter and cardiac muscle are highly anisotropic fibrous tissues with diameters ranging from 0.1–20 μm [1], [2], [3], [4], [5]. In both water diffuses more freely along the dominant fibre orientation and is hindered to different degrees in other directions, leading to diffusion anisotropy [5], [6], [7], [8]. In brain grey matter, which has random microstructures consisting of neuronal cell bodies, neuropil, glial cells, and capillaries, the mobility of water molecules is measurably similar in all directions and is termed isotropic diffusion [9], [10]. The anisotropy of water diffusion in tissue and the sensitivity of water diffusion to the underlying tissue microstructure form the basis for exploiting diffusion magnetic resonance imaging (dMRI) as a non-invasive tool to infer the microstructures of various tissues including brain and heart.

There is an emerging area of research on the development of polymeric materials composed of core-shell structured microfibres that can mimic the microstructure of brain and cardiac fibrous tissues for application in tissue engineering and drug delivery [11], [12], [13], [14] and for brain tumour models [15]. We were the first to use co-electrospun core-shell structured microfibres to construct brain white matter, grey matter and cardiac tissue mimicking phantoms for the calibration and validation of dMRI [5], [16], [17]; this has now been extended to tumour cell-mimicking phantom composed of hollow microspheres [18]. More recently, hollow polypropylene (PP) filaments generated by melt spinning have been also used to mimic white matter axons and to construct an MR brain phantom [19].

Among polymers for biomedical applications, poly(ε-caprolactone) (PCL) is most commonly used for electrospinning for the fabrication of nanofibres as scaffolds, drug delivery systems and medical devices [20], [21] because it has low toxicity, relatively good mechanical properties and can be processed easily, compared with other biodegradable polymers, such as poly(lactide) (PLA), poly(glycolic acid) (PGA), and poly(lactide-*co*-glycolide) (PLGA) [22], [23]. For instance, in our previously developed brain and cardiac phantoms and Rao's tumour model, PCL was used as the shell material in the co-electrospinning process [5], [15], [16], [17], [24].

**Fig. 1:**
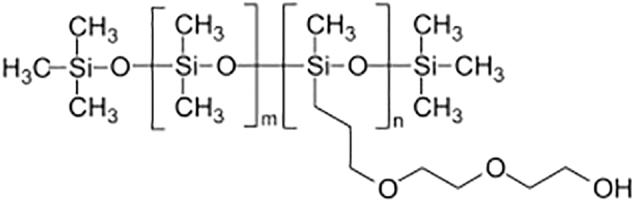
Molecular structure of PSi, a siloxane-based surfactant.

However, PCL is intrinsically hydrophobic, resulting in poor wettability, lack of cell adhesion and uncontrolled biological interactions with the material. There has therefore been long-standing interest in the development of hydrophilic electrospun PCL fibres. Both physical (blending/coating with hydrophilic polymers [25]) and chemical methods (plasma treatment [26], [27], [28], [29], [30], sodium hydroxide treatment [31], [32], grafting or their combination [33], and block copolymers containing PCL [26], [30], [34]) have been employed to improve the wettability of PCL fibres. Among these methods, physical blending of PCL with hydrophilic polymers appears to be the most straightforward. In this work, we investigate the feasibility of using poly[dimethylsiloxane-*co*-[3-(2-(2-hydroxyethoxy)ethoxy) propyl] methylsiloxane] (abbreviated as PSi, Fig. 1), a non-ionic surfactant composed of polyoxyethlyene chains attached to siloxane chains, to enhance the hydrophilicity of PCL fibres.

## 2 Materials and methods

### 2.1 Materials

Polycaprolactone (PCL, number averaged molecular weight Mn = 70 k–90 k), and Polyethylene oxide (PEO, viscosity average molecular weight Mv = 900 k) were obtained from Sigma-Aldrich (Dorset, UK). The additive PSi, was also obtained from Sigma-Aldrich (Cat No. 480320). According to the supplier, PSi has a viscosity of 75 cSt, however the exact values of *m* and *n* in Fig. 1 are not provided. The solvents chloroform (CHCl_3_) and *N*,*N* dimethyl-formamide (DMF) were also purchased from Sigma Aldrich (Dorset, UK). Deionized water or chloroform was used to dissolve the PEO.

### 2.2 Electrospinning of PCL-PSi fibres

PCL microfibres were fabricated using a setup schematically shown in our previous work [35].

The solutions and process parameters for electrospinning/co-electrospinning are given in Table 1. In brief, a mixed solvent of CHCl_3_ and DMF (w/w = 8/2) was used to dissolve PCL at a polymer concentration of 9 wt%. In order to investigate the morphology size and hydrophilicity enhancement of PSi on PCL fibres, 12 different compositions of PCL polymer and PSi surfactant mixtures (from 100/0 to 78/22, w/w PCL/PSi) were prepared. A high-voltage power supply was employed to tune the applied voltage between 0 and 30 kV. A 10 mL plastic syringe with a stainless-steel needle (inner diameter 1.19 mm) mounted on a syringe pump was used to feed PCL solution to the needle tip with a controllable feed rate. The fabricated fibres were then collected on a grounded collector. All experiments were conducted using 2 mL/h flow rate, 15 cm working distance (between the spinneret and fibre collector), 10 kV applied voltage for ~ 10 min in a fume cupboard under ambient conditions.

**Table 1:** Solutions and process parameters used for electrospinning and co-electrospinning.

	Solution	Process parameters
Electrospinning	9 wt% PCL in CHCl_3_ + DMF (8/2 w/w) with 0–22 wt% PSi	10 kV applied voltage 2 mL/h flow rate 15 cm working distance
Co-electrospinning	Shell 9 wt% PCL in CHCl_3_ + DMF (8/2 w/w) with 1 wt% or 0.4 wt% PSi	16 kV applied voltage 3 mL/h shell flow rate 16 cm working distance
	Core 4 wt% PEO in distilled water 2.5 wt% PEO in CHCl_3_	0.25 to 4.0 mL/h core flow rate 0.25 to 1 mL/h core flow rate

### 2.3 Co-electrospinning of shell-core PCL-PSi -PEO microfibres

In a typical procedure for co-electrospinning, a mixing solution of 9 wt% PCL in CHCl_3_/DMF with 1 wt% PSi was used as the shell solution and PEO in deionized water or chloroform acted as the core solution. The co-electrospinning was carried out on a lab-scale electrospinning setup described in our previous publication [35]. All experiments were conducted in a fume cupboard at ambient conditions. To investigate the effect of core flow rate on the morphology of co-electrospun fibres, the shell flow rate was set at 3 mL/h. For PEO/water and PEO/CHCl_3_ core solution, the flow rate was varied from 0.25 to 4.0 mL/h and from 0.25 to 1 mL/h, respectively. Other co-ES parameters were as follows (unless stated otherwise): applied voltage of 16 kV, working distance of 16 cm. The resultant fibres were then collected on a grounded static metal plate or a rotating drum. Once collected the inner core solution evaporates, leaving a solidified outer sheath and therefore hollow fibres.

### 2.4 Characterization of electrospun and co-electrospun PCL microfibres

The surface morphology and cross sections of electrospun and co-electrospun fibres were observed using a Philips XL30 FEG SEM and G2 pro SEM with an accelerating voltage of 5 kV. Fibre specimens were coated with a platinum film with a thickness of approximately10 nm to increase their conductivity. For imaging of fibre cross sections, fibres were cut by sharp scissors in liquid nitrogen. Image processing software ImageJ (NIH) was used to measure the fibre inner diameters from the SEM micrographs. For each sample, fibre inner diameters were measured at 20 different points within SEM images to determine the mean values and standard deviations.

### 2.5 Wettability of electrospun and co-electrospun PCL microfibres

The wettability of electrospun/co-electrospun PCL microfibres was evaluated using the Krϋss DSA 100 Drop Size Analyzer (Krüss GmbH, Hamburg, Germany). Fibres were deposited on glass slides for at least 10 min. A 10 μL droplet of distilled water was released onto each sample using a 500 μL syringe (Hamilton, Switzerland) and 1.991 mm needle (Krϋss GmbH, Hamburg, Germany). The evolution of the drop profile (contact angle, drop radius, drop volume) was recorded over time using high speed imaging and used to demonstrate the absorption of water by fibre substrates. The analyzer provides high-precision dosing and positioning of liquid drops and permits recording and evaluation of video images through accompanying PC controlled software. The PSi composition of electrospun/co-electrospun fibres was characterized by X-ray energy dispersive spectroscopy (EDX) attached to Quanta FEG 650 instrument operating at 5 kV.

### 2.6 Phantom preparation

Co-electrospun fibres were packed into glass tubes that were filled with diffusion liquid – deionized water, using the method described in our previous work [16]. Two sets of phantoms were prepared for hydrophilic testing from co-electrospun PCL and PCL-PSi fibres, respectively.

### 2.7 MR imaging

Co-electrospun fibre phantoms were immersed in water for about one week before MR scan. Diffusion tensor imaging using a pulsed gradient spin-echo with 30 gradient directions, b = 800 s mm^− 2^ (plus 1 b = 0 s mm^− 2^), δ = 4 ms, Δ = 10 ms, G_max_ = 302.8 mT m^− 1^ was carried out on a Bruker 7 T horizontal bore magnet (Bruker Biospin, Germany). Other sequence parameters were: axial FOV 3 cm × 3 cm, 128 × 128 matrix, total of 10 slices with 1 mm slice thickness, TR = 5 s, TE = 20.5 ms. The MR signal comes from water that diffuses within the fibres. Room temperature was monitored to be 24.1 °C and was found to vary by a maximum of 0.7 °C within a given scan session.

### 2.8 Statistical analysis

The data on fibre diameter were not all normally distributed and were analysed using Kruskal–Wallis ANOVA (Origin Pro, version 9, OriginLab, Northampton, MA). Statistical significance was accepted at *p* < 0.05.

## 3 Results

### 3.1 Electrospinning of PCL-PSi nanofibres

In this study, PSi was chosen due to its high hydrophilicity. The content of PSi in 9 wt% PCL/CHCl_3_ + DMF (8/2) solution was varied from 0 to 22 wt% to investigate its effect on the process stability, morphology, size and wettability of resultant fibres. The electrospinning process was operating for ~ 10 min using the following settings: applied voltage 10 kV, working distance 15 cm and flow rate 2 mL/h. These parameter settings were chosen based on the observation of a stable fluid jet present in the electrospinning process.

**Fig. 2:**
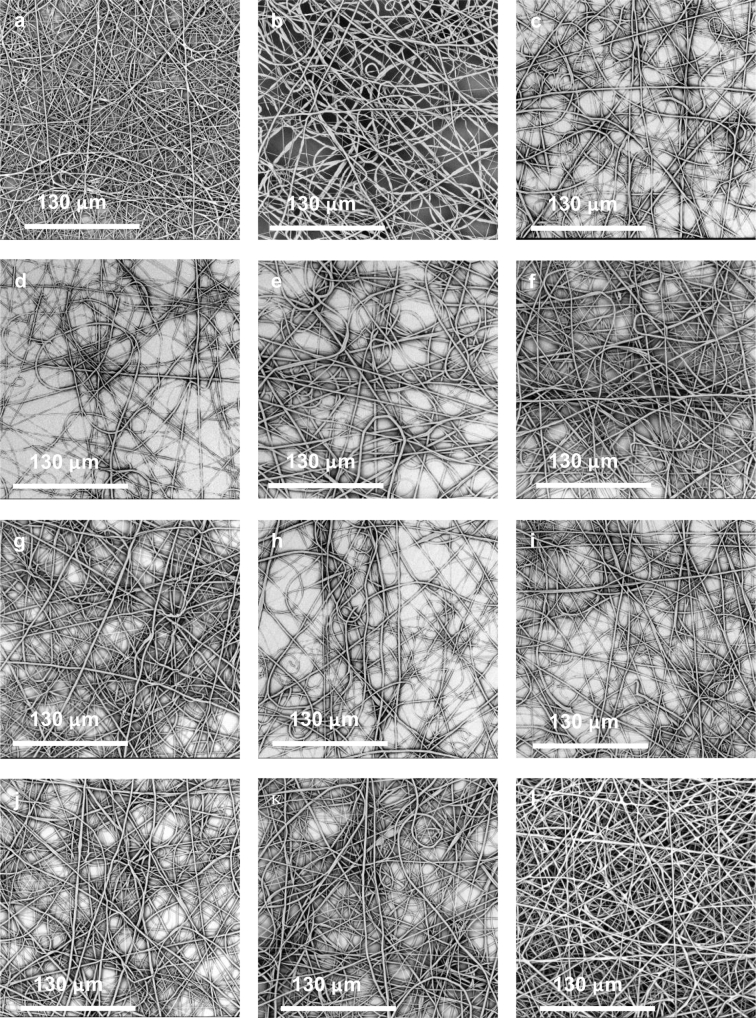
Electrospinning of PCL-PSi nanofibres with different PSi concentrations. (a) 22 wt%, (b) 18 wt%, (c) 14 wt%, (d) 10 wt%, (e) 6 wt%, (f) 2.2 wt%, (g) 1.2 wt%, (h) 1.0 wt%, (i) 0.8 wt%, (j) 0.6 wt%, (k) 0.4 wt% (l) 0 wt%.

**Fig. 3:**
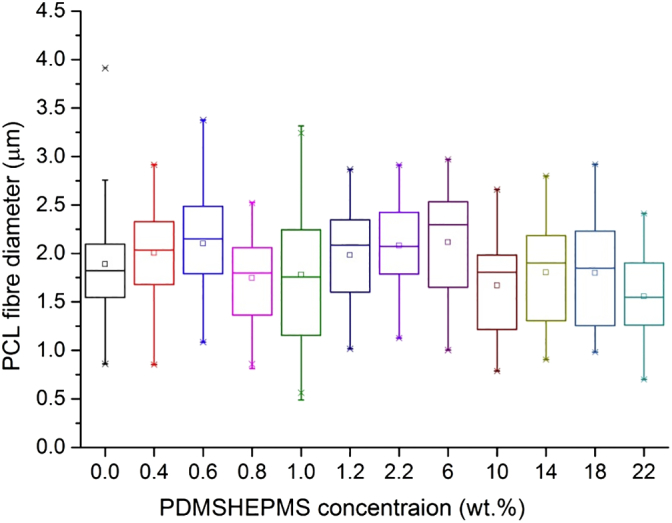
Box-plot of size distribution of electrospun fibres obtained from PCL/CHCl_3_ + DMF solution with 0 to 22 wt% PSi.

The results obtained by SEM analysis, as shown in Fig. 2, suggest that all electrospun PCL-PSi fibre appeared bead-free and there were no obvious changes in morphology of the resultant fibres with the decreasing content of PSi from 22 wt% to 0 wt%, indicating little effect of the addition of PSi on the fibre formability of the PCL solution. SEM micrographs with × 5 k mag (not shown) further reveal that these electrospun fibres were not uniform and were significantly variable (KWANOVA, *p* < 0.05) in diameter ranging from 0.5 to 3.5 μm. However, there are no clear trends in either median or mean diameters of these fibres with the changing content in PSi content, as shown by Fig. 3.

### 3.2 Co-electrospinning of PCL and PCL-PSi with PEO

#### 3.2.1 Co-electrospinning of PCL-PSi with PEO/water

**Fig. 4:**
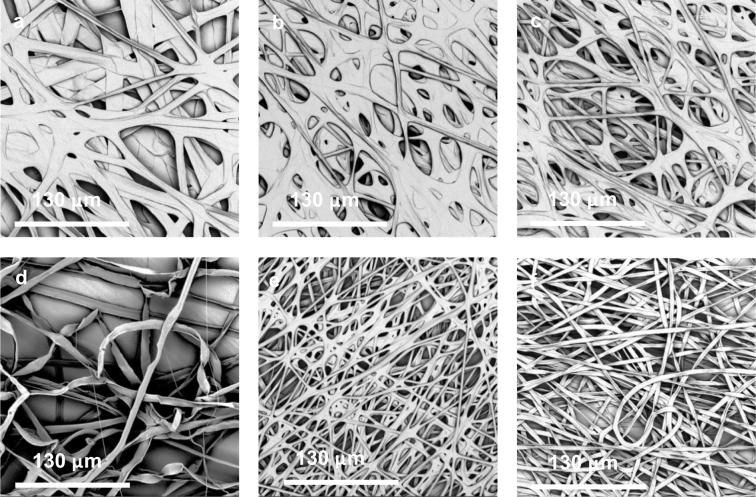
SEM micrographs showing the morphology of co-electrospun fibres from PCL-PSi shell and PEO/water core using different **core flow rates**. (a) 4 mL/h, (b) 3 mL/h, (c) 2 mL/h, (d) 1 mL/h, (e) 0.5 mL/h, (f) 0.25 mL/h.

**Fig. 5:**
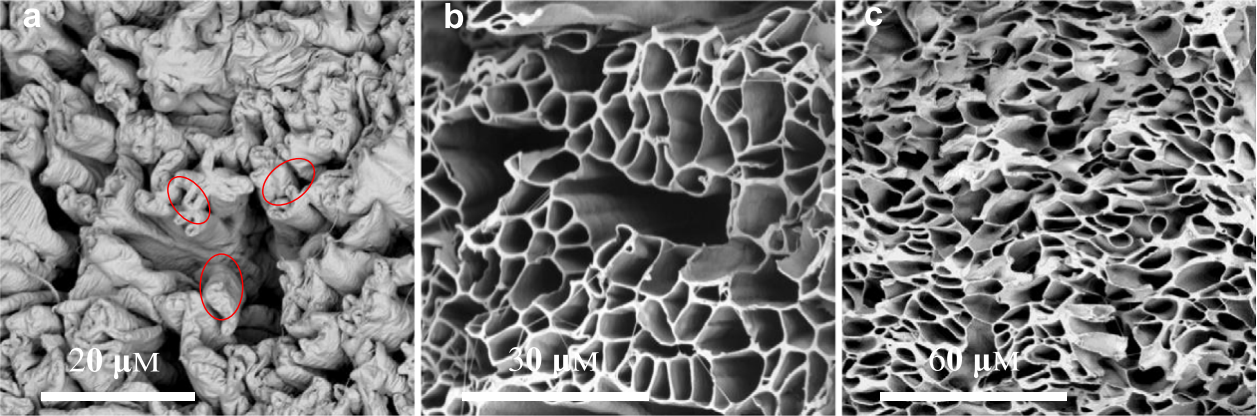
SEM micrographs showing the morphology of co-electrospun fibres from PCL-PSi shell and PEO core. (a) 9 wt% PCL/CHCl_3_ + DMF(8/2, w/w) with 1 wt% PSi as shell, 4 wt% PEO/water, applied voltage 9 kV, working distance 15 cm, shell/core flow rate 3/1 mL/h, ~ 6 h operation; (b) 9 wt% PCL/CHCl_3_ + DMF(8/2, w/w) as shell, 4 wt% PEO/water, applied voltage 9 kV, working distance 5 cm, shell/core flow rate 3/1 mL/h, ~ 30 min operation; (c) ~ 6 h operation.

**Fig. 6:**
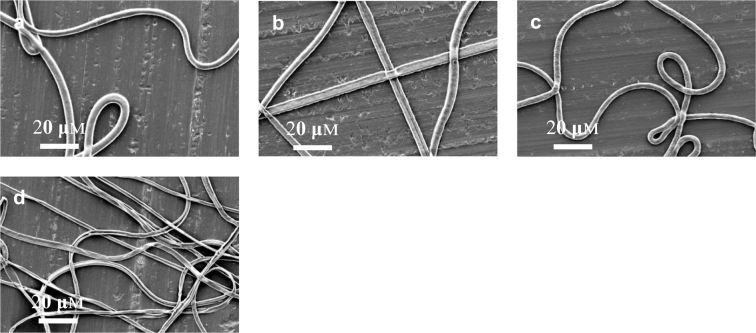
SEM micrographs showing the morphology of co-electrospun fibres from PCL shell and PEO or sugar/water core using different **process parameters**. (a) PCL/CHCl_3_ + DM – PEO/water, 18.0 kV applied voltage, **24 cm** working distance, 3.0/0.2 mL/h shell/core flow rate; (b) PCL/CHCl_3_ + DMF – PEO/water,18.0 kV applied voltage, **16 cm** working distance, 3.0/0.2 mL/h shell/core flow rate; (c) PCL/CHCl_3_ + DMF – sugar/water,18 kV applied voltage, 20 cm working distance, **3.0**/0.2 mL/h shell/core flow rates; (d) PCL/CHCl_3_ + DMF-sugar/water, 18.0 kV applied voltage, 20 cm working distance, **1.0**/0.2 mL/h.

Based on Section 3.1, a very small amount PSi (i.e., 1 or 0.4 wt%) was added into the shell solution to minimize its effect on PCL solution spinnability and process stability while keeping the hydrophilic property of resulted PCL-PSi fibres. Here 1 wt% PSi was added into 9 wt% PCL in CHCl_3_/DMF(4:1, w/w) used as the shell solution. The core solution was PEO in water with 4.0 wt% concentration and was chosen because PEO has good electrospinnability and can help achieve a stable process. Coaxial electrospinning of random fibres was carried out using the following parameters: 9.0 kV applied voltage, 23 cm working distance, shell flow rate 3 mL/h and core flow rate was varied from 4 to 0.25 mL/h. With the addition of 1 wt% PSi in the PCL shell, a stable compound jet, which was usually seen in co-electrospinning of neat PCL and PEO solutions, was not achieved due to the fact the solution droplet on the needle tip dripped regularly over a range of applied voltage and/or flow rate. Solution dripping became less frequent with the decreasing core flow rate. As shown by SEM micrographs in Fig. 4, decreasing core flow rate rendered a morphological change from film-like structures (where fibres fused) to random fibre mesh. The resultant fibres from the combination of PCL-PSi shell and PEO/water core appeared completely collapsed, resulting in flat belts regardless of the core flow rate, which were not present in the electrospinning of non-hollow PCL-PSi fibres (Fig. 2). SEM micrographs in Fig. 5 show the cross-sectional structures of co-electrospun PCL-PSi and PCL shells with a PEO core. It is clearly shown that PCL-PSi fibres in Fig. 5a were collapsed but porous cross-sectional structures were present in PCL fibres in Fig. 5b and c. Fibres with a circular cross section with a smooth surface can be produced from PCL shell and PEO/water core in appropriate core/shell flow rates (Fig. 6a), indicating the introduction of PSi in PCL shell solution is likely to be responsible for the formation of collapsed fibres. These observations demonstrate that the shell/core polymer solutions play an important role in the formation of fibre cross-section shape. It should be pointed out that inappropriate process parameters can also result in collapsed fibres from PCL shell and PEO core (Fig. 6b). A similar phenomenon was also observed in the co-electrospinning of PCL shell and sugar core. In paricular, a decrease in the flow rate of the PCL shell soution from 3 mL/h, where the fibres have a circular cross section (Fig. 6c), to 1 mL/h, resulted in the formation of flat fibres (Fig. 6d).

#### 3.2.2 Co-electrospinning of PCL-PSi with PEO/CHCl_3_

**Fig. 7:**
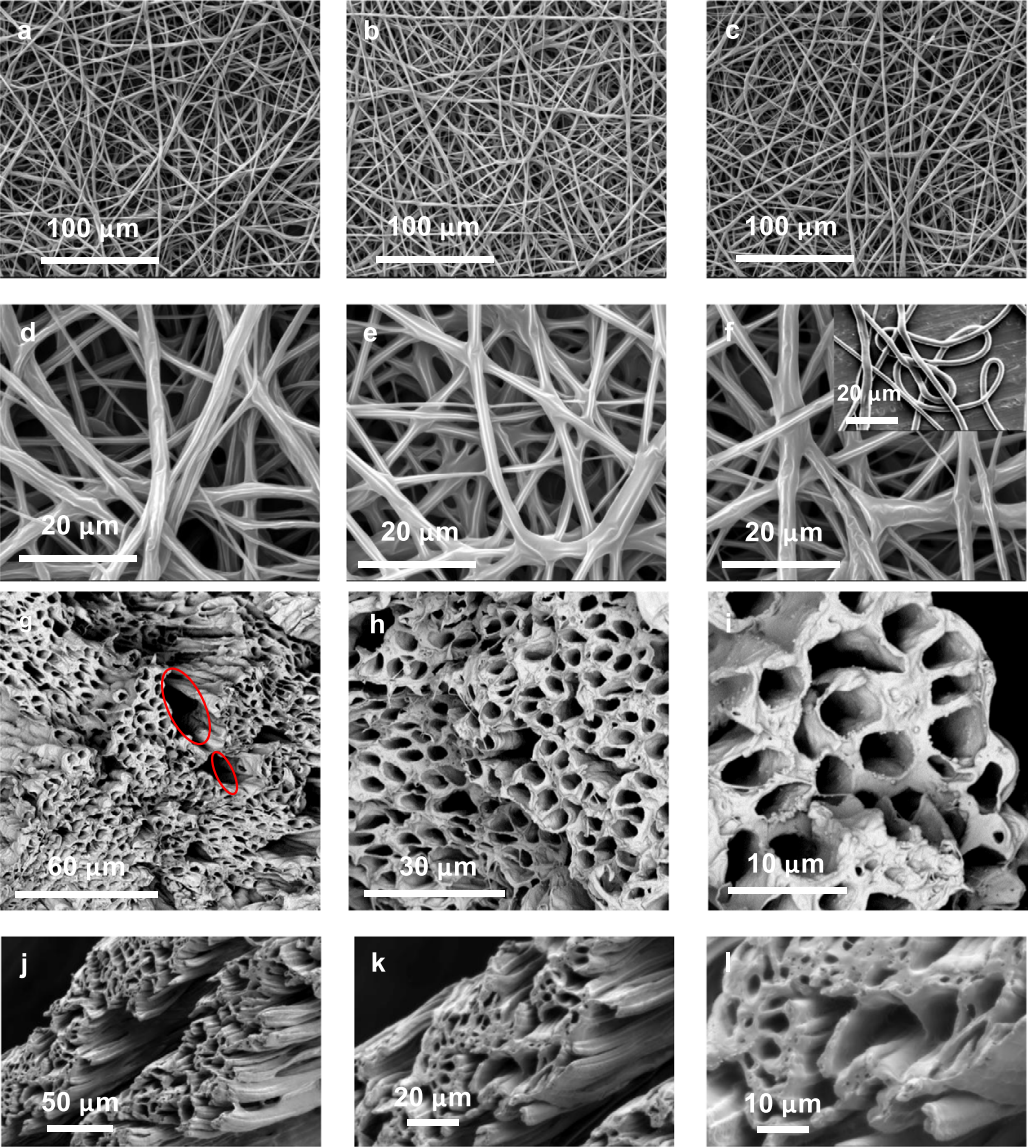
SEM micrographs of co-electrospun random fibres from PCL-PSi shell and PEO/CHCl_3_ core with various core flow rates 1.0 mL/h (a–d), 0.75 mL/h (b–e) and 0.5 mL/h (c–f) and aligned fibre bundles with 1 wt% PSi (g–i) and 0.4 wt% PSi (j–l).

In this section, PEO/CHCl_3_ with 2.5 wt% concentration was used as core solution. Random fibres were prepared with the varying core flow rate from 1 mL/h to 0.5 mL/h while the shell flow rate was kept at 3 mL/h, applied voltage at 16 kV and working distance at 23 cm. SEM images at × 2 k magnification (Fig. 7a–c) show the improved morphological change with flow rate when PCL/CHCl_3_ as core rather than PCL/water. The higher process stability of co-electrospinning of PCL-PSi/CHCl_3_ + DMF shell and PEO/CHCl_3_ core contributed to this change. SEM images with × 5 k magnification (Fig. 7d–f) further revealed that fibres had wrinkled surface topographies in all three samples. However, co-electrospun PCL fibres using PEO/water as core show a smooth surface, as shown in the inset of Fig. 7f.

For imaging of the fibre cross sections, uniaxially aligned fibre bundles were collected for about 1 h on a rotating mandrel following the approach outlined in [36], and the oriented fibres were cut by sharp scissors in liquid nitrogen. A fibre bundle was prepared from PCL/CHCl_3_ + DMF with 1 wt% PSi shell and PEO/CHCl_3_ core using shell/core flow of 3.0/0.5 mL/h, applied voltage 14 kV, working distance 10 cm. SEM images of the cross section of these electrospun fibres reveal that the resulting fibres are hollow with a relatively thin, uniform shell and large, hollow core (see Fig. 7g–i). The outer diameter of the tubes is several micrometres while the thickness of the walls is a few hundreds of nanometres. Another fibre bundle was produced from the concentration of 0.4 wt% PSi using the following settings of 3.0/1.0 mL/h, 10.9 kV, 23 cm. SEM images (Fig. 7j–l) reveal fibres in the strip remained hollow but had thicker walls; fibres tended to separate into layers (Fig. 7j).

### 3.3 Water wettability on electrospun PCL-PSi fibres

**Fig. 8:**
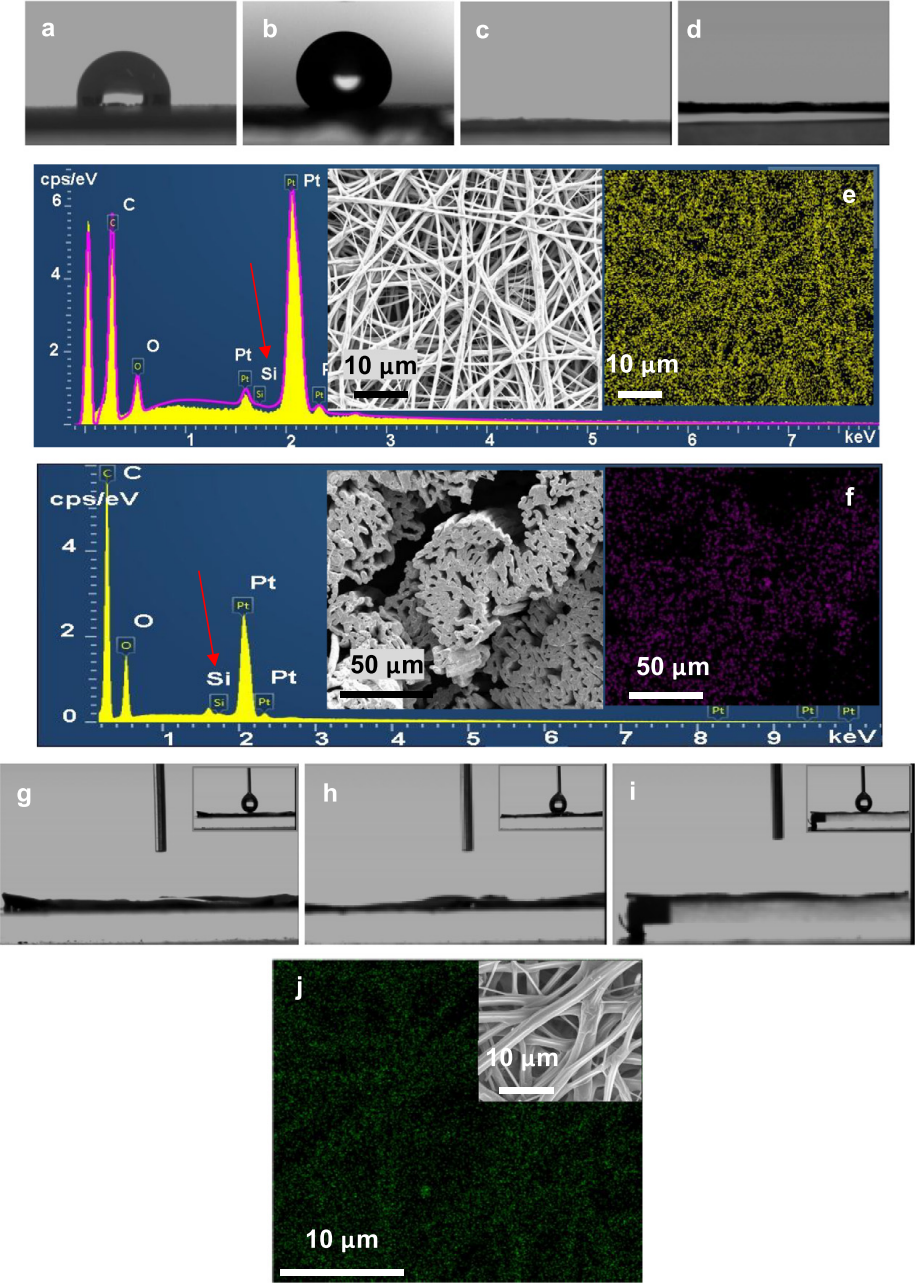
Water wettability of ES/co-ES PCL nanofibres. (a) PCL cast film; (b) Electrospun PCL fibres; (c) Electrospun PCL-PSi-1 wt% fibres; (d) Co-ES PCL-PSi/PEO (1 wt%) fibres; (e) EDX measurement on **surface** of co-ES PCL-PSi sample in (d), inset: SEM of fibre surface and Si distribution on fibre surface; (f) EDX measurement of **cross section** of 10 wt% PCL/CHCl_3_ with 0.4 wt% PSi shell, PEO/CHCl_3_ (2.5 wt%) as core, inset: SEM of cross section and Si distribution on cross section; (g) Electrospun PCL-PSi-0.4 wt% fibres after about one month, inset: initial water drop; (h) Electrospun PCL-PSi-1 wt% fibres after about one month; (i) Co-ES PCL-PSi/PEO (1 wt%) fibres after about one month. Insets in (g-h): water droplet immediately before contacting fibres; (j) EDX measurement showing Si distribution on **surface** of electrospun PCL-PSi-1 wt% fibres after ~ 12 months in water (inset: SEM of fibre surface).

The contact angle of PCL cast film and electrospun PCL fibres was shown to be 91.7 ± 0.2.28° and 129.9 ± 2.13°, respectively (Fig. 8a–b). When 1.0 wt% PSi was added to PCL solution, a water droplet spread completely on the resultant electrospun PCL fibres completely within a few seconds (Fig. 8c), indicating that PCL fibres become highly hydrophilic. PCL solution with 1.0 wt% PSi was used as shell in co-electrospinning with PEO/CHCl_3_ as core, the resultant fibres was also found to be highly hydrophilic, as shown by Fig. 8d. EDX analysis shows the presence of PSi on both the surface of co-electrospun PCL-PSi-1 wt% fibres and the cross-section of co-electrospun PCL-PSi-0.4 wt% fibres. After one-month immersion in water, complete water spreading was still seen across the surface of electrospun PCL-PSi-0.4 wt% (Fig. 8g), PCL-PSi-1 wt% (Fig. 8h) and Co-ES PCL-PSi/PEO (1 wt%) fibres (Fig. 8i), indicating the stability of the PSi in PCL fibres. This was further confirmed by the fact that the PSi element was present in PCL-PSi-1 wt% (Fig. 8j) after immersed in water for 12 month.

### 3.4 MR measurements

**Fig. 9:**
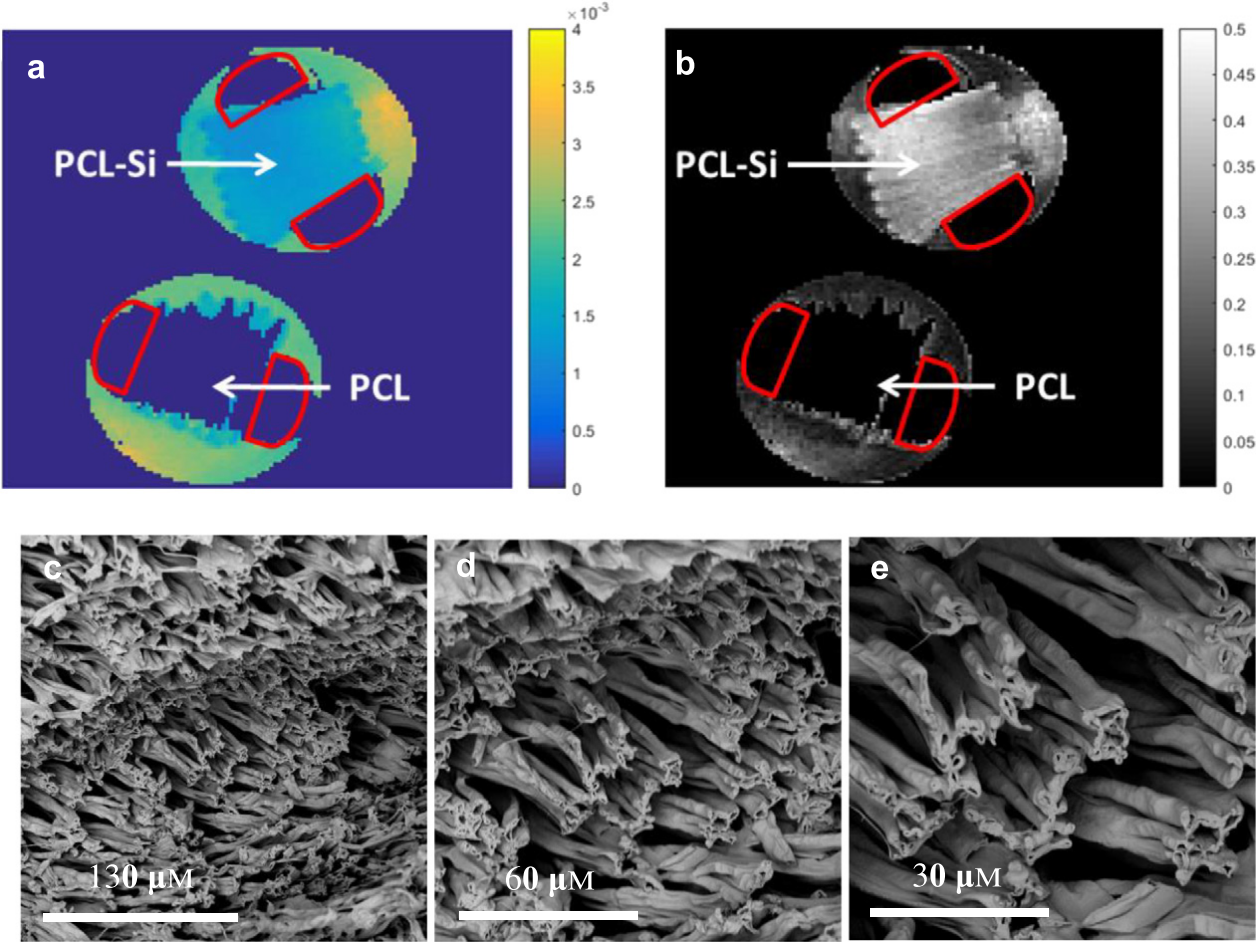
MR scans of PCL-PSi and PCL fibre phantoms. (a–b) MD and FA maps from PCL-PSi-1 wt% (9 kV, 15 cm, 3/1 mL/h, 4 wt% PEO/water as core) and PCL-only fibre phantoms (10 kV, 20 cm, 3.0/0.8 mL/h, 4 wt% PEO/water as core). The red outline depicts the plastic rods used to squeeze the fibre strips together; (c) SEM micrographs with × 1 k, × 2 k and × 4 k magnifications showing cross-section of PCL-PSi-1.0 wt% phantom. (For interpretation of the references to colour in this figure legend, the reader is referred to the web version of this article.)

Co-electrospun fibre strips PCL and PCL with 1.0 wt% PSi were used to make two water-filled phantoms using the method previously described [24]. The parameter maps were masked to regions to delineate the phantom from the surrounding free water and a regions-of-interest (ROI) were found in the phantom layers squeezed together by the plastic rods, as shown on the mean diffusivity (MD) and fractional anisotropy (FA) maps in Fig. 9a–b. It can be seen from the MD map (Fig. 9a) that strong MR signal was detected in 1 wt% PCL-PSi phantom, but not in the PCL-only phantom, indicating that water can only penetrate PCL-PSi phantoms. The FA map (Fig. 9b) reveals the fibre orientation in 1 wt% PCL-PSi phantom is anisotropic. The SEM image of the 1 wt% PCL-PSi fibres shows the parallel fibre orientation (Fig. 9c–e).

## 4 Discussion

Highly hydrophilic surfaces are those that exhibit water contact angle close to 0° and can be achieved by adding strong polar group to the surface [37]. PSi is a water-soluble polysiloxane surfactant having a large number of polar hydroxyl groups (—OH). The use of PSi could result in size variation of PCL fibres but did not appear to impart any adverse effect on the electrospinning process of PCL.

The reason for this variation is currently unclear as the relationship between fibre size and PSi concentration was not established. However, we assume at least two factors could have contributed to the variation in fibre diameters. First, as indicated by molecular structures of CHCl_3_ and PSi, they possess very different solution properties, such as viscosity (PSi 75 cP vs CHCl_3_ 0.537 cP at 25 °C, from Sigma), although other relevant information (electric conductivity and surface tension) on PSi is not readily available. The addition of the PSi component into PCL/CHCl_3_ + DMF increased the shell solution viscosity, which could result in the size difference of electrospun PCL/PSi fibres. Second, it is widely accepted that the electrospinning process can be affected by environment conditions such as humidity and temperature. Our current electrospinning system doesn't allow us to control these conditions during its operation, which could also contribute to the fibre size variation [38], [39].

No obvious effect of PSi on the electrospinning process was highly desired by a well-stabilized co-electrospinning of PCL-PSi shell with PEO core and well-defined core-shell structured fibres. In our previous studies, a stable process was often achieved from the shell/core combination of PCL in CHCl_3_/DMF (8:2, w/w)/PEO in water using a relatively wide range of applied voltage and core/shell flow rate, which resulted in well-defined hollow PCL microfibres [16], [24], [36]. It has also been previously reported that cross-sectional shape of electrospun fibres can be affected not just by the polymer/solvent properties but also by the process parameters [40], [41], [42]. The formation of collapsed PCL fibres in the co-electrospinning of PCL-PSi in CHCl_3_/DMF shell with PEO in water core can be readily explained as follows: the presence of hydrophilic PSi hampers the evaporation of water in the core solution via the shell and thus delays the solidification of PCL shell that plays a key role in the formation of hollow PCL microfibres [40].

Co-electrospun hollow PCL microfibres have been successfully employed as building block to construct fibrous tissue mimicking phantoms. In those developed PCL phantoms, an organic solvent (cyclohexane) had to be used as diffusion liquid for MR measurement due to the hydrophobic property of PCL. These cyclohexane-filled phantoms have demonstrated the excellent MR signal, MR reproducibility and chemical stability [16], [43]. However, the use of cyclohexane has caused several practical problems including liquid refilling (at least once a week) due to evaporation and potential hazards leading to complicated packing for transporting phantoms in multi-centre studies. Due to the above-mentioned reason, hollow microfibres with a circular cross section could not be produced from the shell and core combination of PCL + PSi/CHCl_3_ + DMF and PEO/water. The PEO/CHCl_3_ was used as core solution to replace the PEO/water and was found to help stabilize the co-electrospinning process and thus produce PCL fibres with a circular cross section and surprisingly wrinkled surface. Wang et al. proposed a mechanism for the formation of wrinkled fibres that result from buckling instabilities during electrospinning [44]. This explanation can also apply to the wrinkled co-electrospun PCL fibres observed here. During the co-electrospinning process, The PEO/CHCl_3_ core was much more volatile than the PEO/water core, which resulted in its much faster evaporation through the thin, elastic glassy PCL shells that first solidified. The PEO/CHCl_3_ core therefore shrank more quickly than PEO/water in the PCL shell, which was more likely to result in a contraction mismatch between the core and PCL shell (Fig. 7). Based on the mechanism proposed by Wang et al., this contraction can facilitate wrinkle formation on the surface of co-electrospun PCL fibres.

More interestingly, hollow microfibres were formed in one-step in the co-electrospinning of PCL + PSi/CHCl_3_ + DMF and PEO/CHCl_3_ and most of the fibres merged together but still preserved their cylindrical shape without any catastrophic collapse, which indicates that the walls, although relatively thin, are sufficiently robust. The PEO core was not dissolved but is expected to be deposited as a very thin layer (~ 100 nm) onto the inner surface of hollow PCL microfibres [40]. Similar results were previously obtained with other two shell/core combinations in which PCL/CHCl_3_ + DMF was shell and PEO/water or sugar/water was used core [24], [35], [36]. It has to be mentioned that the formation of cross-sectional pores and porosity of co-electrospun fibres were influenced by both process parameters and collecting methods, but less controlled than those individual hollow microfibres [36].

The difference in contact angle could be explained by the fact that PCL is hydrophobic and PCL fibres have rougher surface than PCL cast film. It is well known that if a polymer is hydrophobic, increasing its roughness causes an increase in the hydrophobicity of this polymer; on the other hand, if the polymer is hydrophilic, its hydrophilicity is also found to increase with increasing surface roughness. We envisage that the potential molecular chain entanglements between PCL and PSi may account for the persistent hydrophilic surfaces of PCL-PSi fibres for 12 months. However, PSi is not used in pharmaceuticals or food preparation and relatively little information is available regarding its stability and degradation [45]. Nevertheless, PSi has two units of ethylene oxide in its hydrophilic section, which are likely to be vulnerable to autoxidation. The polysiloxane section, however, is rather inert, with oxidation producing silicon dioxide. We have to mention that it would be highly desirable for electrospun PCL fibres to blend with hydrophilic and biocompatible additives like natural lecithin [46] or synthetic poly(vinyl alcohol) (PVA) [25] for their application in tissue engineering, though the biocompatibility isn't so critical as water wettability for MR phantoms in the present study.

## 5 Conclusion

In this study, co-electrospun PCL-PSi fibres were successfully applied in the field of tissue-mimicking phantoms filled with water for diffusion magnetic resonance imaging. The concentration of PSi had a significant effect on the size of PCL-PSi fibres while all fibres had smooth surface morphology. When PCL-PSi solution was used as shell fluid for the co-electrospinning with PEO/water core, a stable process was not achieved, resulting in the formation of collapsed fibres; when PEO/CHCl_3_ was used as the core fluid, the co-electrospinning process became much more stable, from which well-defined and hollow fibres were produced. However, the resultant fibres had wrinkled surface topographies due to the contraction mismatch between the core and shell during solvent evaporation. The water wettability of electrospun and co-electrospun PCL-PSi fibres was evaluated by surface contact angle. The addition of PSi into PCL solution enabled PCL-PSi fibres to gain the value of 0° for the water contact angle test. These finding showed the PCL-PSi fibres are highly hydrophilic. Even after immersing in water for a time period of up to 12 months, the highly hydrophilic property of PCL-PSi fibres remained. EDX analysis confirmed the presence of Si on both the PCL fibres surface and cross-sections, which is believed to be responsible for the water wetting stability. Co-electrospun PCL and PCL-PSi hollow fibres were constructed into water-filled phantoms. MR measurement reveals that the motion of water molecules was detected by dMRI only from PCL-PSi phantom and anisotropic. Co-electrospinning of PCL-PSi with PEO/CHCl_3_ is a feasible approach producing hollow microfibres with highly hydrophilic property. The highly hydrophilic hollow microfibres have desirable properties for the construction of brain and cardiac tissue-mimicking water-filled phantoms.
